# Behavioral and Cellular Effects of Vitamin A Deficiency in a J20:RARE‐LacZ mouse model of Alzheimer's Disease

**DOI:** 10.1002/alz70855_106048

**Published:** 2025-12-24

**Authors:** Adam Baker, Ashly Hindle, Chhanda Bose, Shane Smith, Shadt Skawratananond, J. Josh Lawrence

**Affiliations:** ^1^ Garrison Institue on Aging, Lubbock, TX, USA; ^2^ Texas Tech University Health Sciences Center, Lubbock, TX, USA; ^3^ Center for Translational Neuroscience and Therapeutics, Lubbock, TX, USA

## Abstract

**Background:**

All‐trans retinoic acid (ATRA), a metabolite of vitamin A (VA), has been shown to upregulate ADAM10, an α‐secretase enzyme responsible for non‐amyloidogenic APP cleavage by binding to promoter retinoic acid response elements (RARE). This finding has led to investigations into VA derivatives as potential therapies for Alzheimer's Disease (AD), but it is unclear whether nutritional VA deficiency exacerbates AD pathology. Originally used to map embryonic retinoic acid (RA) signaling, RARE‐LacZ mice possess multiple LacZ genes controlled by RAREs. In this pilot study, RARE‐LacZ mice were crossed with the J20 mouse model of AD to study the effects of chronic dietary VA deficiency on learning and memory, as well as ATRA signaling within the hippocampal dentate gyrus (DG).

**Method:**

Dietary manipulation of VA was achieved using standard AIN‐93M diet (4 IU/g VA) and a modified VA‐deficient (0.4 IU/g) AIN‐93M diet. Mice were weaned onto experimental diets and behaviorally tested at 9‐10 months of age using open‐field, novel object location/recognition, water T‐maze, and rotarod tests. Mice were sacrificed within 24 hours following the last day of testing. Brain hemispheres were preserved in PFA for immunohistochemical analyses.

**Result:**

Immunohistochemical findings suggest Aβ plaques within the hippocampus disrupt DG integrity and may interfere with ATRA signaling in DG granule cells. In the water T‐maze, wild‐type sibling littermates J20^‐/‐^:LacZ^+/‐^ mice (*n* = 21) displayed an accelerated learning ability and stronger memory of the platform location compared to J20^+/‐^:LacZ^+/‐^ mice (*n* = 24), as measured by mean latency to platform and time spent in the original platform arm location after the platform was moved. Diet did not appear to have a significant effect on either genotype. Analysis of performance in the open field and novel object tests is currently ongoing.

**Conclusion:**

J20^+/‐^:LacZ^+/‐^ mice aged 9‐10 months display Aβ aggregation throughout the hippocampus and cortex, which may be responsible for local changes in ATRA signaling in the DG. Ongoing behavioral data analysis will give us additional information about the correlation between diet, behavioral performance, and AD pathology in the DG.

